# Phylogenetic and recombination analysis of *Tobacco bushy top virus* in China

**DOI:** 10.1186/s12985-015-0340-2

**Published:** 2015-07-25

**Authors:** Deya Wang, Chengming Yu, Guolu Wang, Kerong Shi, Fan Li, Xuefeng Yuan

**Affiliations:** College of Plant Protection, Shandong Agricultural University, Tai’an, 271018 People’s Republic of China; College of Animal Science and Veterinary Medicine, Shandong Agricultural University, Tai’an, 271018 People’s Republic of China; Key Laboratory of Agricultural Biodiversity for Pest Management of China Education Ministry, Yunnan Agricultural University, Kunming, 650201 People’s Republic of China

**Keywords:** TBTV, RACE, Phylogenetic analysis, Recombination

## Abstract

**Background:**

During the past decade, tobacco bushy top disease, which is mainly caused by a combination of *Tobacco bushy top virus* (TBTV) and *Tobacco vein-distorting virus* (TVDV*)*, underwent a sudden appearance, extreme virulence and degeneration of the epidemic in the Yunnan province of China. In addition to integrative control of its aphid vector, it is of interest to examine diversity and evolution among different TBTV isolates.

**Methods:**

5’ and 3’ RACE, combined with one step full-length RT-PCR, were used to clone the full-length genome of three new isolates of TBTV that exhibited mild pathogenicity in Chinese fields. Nucleotide and amino acid sequences for the TBTV isolates were analyzed by DNAMAN. MEGA 5.0 was used to construct phylogenetic trees. RDP4 was used to detect recombination events during evolution of these isolates.

**Results:**

The genomes of three isolates, termed TBTV-JC, TBTV-MD-I and TBTV-MD-II, were 4152 nt in length and included one distinctive difference from previously reported TBTV isolates: the first nucleotide of the genome was a guanylate instead of an adenylate. Diversity and phylogenetic analyses among these three new TBTV isolates and five other available isolates suggest that ORFs and 3’UTRs of TBTV may have evolved separately. Moreover, the RdRp-coding region was the most variable. Recombination analysis detected a total of 29 recombination events in the 8 TBTV isolates, in which 24 events are highly likely and 5 events have low-level likelihood based on their correlation with the phylogenetic trees. The three new TBTV isolates have individual recombination patterns with subtle divergences in parents and locations.

**Conclusions:**

The genome sizes of TBTV isolates were constant while different ORF-coding regions and 3’UTRs may have evolved separately. The RdRp-coding region was the most variable. Frequent recombination occurred among TBTV isolates. Three new TBTV isolates have individual recombination patterns and may have different progenitors.

**Electronic supplementary material:**

The online version of this article (doi:10.1186/s12985-015-0340-2) contains supplementary material, which is available to authorized users.

## Introduction

*Tobacco bushy top virus* (TBTV) is a member of the *Umbravirus* genus, which requires the presence of *Tobacco vein-distorting virus* (TVDV*)* for infectivity in the field [1]. TBTV is encapsidated by the coat protein encoded by TVDV, which is needed for transmission via aphids [1, 2]. However, mechanical inoculation of sap from diseased tobacco onto healthy plants leads to the loss of TVDV, implying the independent pathogenicity of TBTV [1]. Together, these viruses caused severe stunting and destructive bushy-top disease in tobacco in sub-Saharan Africa in the 1960s [3] and in Asia including China in the 1990s [1]. During 1993 to 2001, there were 51,300 hm^2^ of tobacco bushy top-diseased fields including 8,700 hm^2^ of total field failure in Yunnan Province of China [4]. In addition to TBTV and TVDV, two components including tobacco bushy top disease-associated RNA (TBTDaRNA) and satellite RNA of TBTV were also identified in tobacco with bushy-top disease [5, 6], although all four components were not always present together [7].

During the past decade, tobacco bushy-top disease was infrequent with only sporadic cases exhibiting mild symptom in Yunnan province, which may be due to interruption of the natural epidemic cycle through the integrative control of its aphid vectors [7, 8]. Sudden appearance, extreme virulence and degeneration of the epidemic of tobacco bushy-top disease was a pattern similar to that of other destructive diseases [9–12], whose lethal pathogens underwent quick attenuation of pathogenicity. Therefore, it is of interest to determine whether the new TBTV isolates produced mild pathogenicity and how they were evolved. For single-stranded RNA viruses, recombination is a major evolutionary event allowing isolates to adapt to new environmental conditions and hosts [13], and frequent recombination events have been detected for various RNA viruses such as *Soybean mosaic virus* and potyvirus isolates [14–16].

The TBTV genome contains a positive-sense single-stranded RNA of 4152 nt, which encodes four ORFs, and contains a short 5’ UTR of 10 nt and a 3’ UTR of 645 nt [17]. Based on comparisons with other *Umbraviruses*, p35 and its frameshift product p98 are responsible for genome replication [18–20]. p98 contains the ubiquitous RdRp GDD motif of positive-strand RNA virus and is presumably the RNA-dependent RNA polymerase (RdRp) [18, 21]. Based on studies conducted with *Groundnut rosette umbravirus,* p26 is a long-distance movement-associated protein and is also responsible for stabilization of viral RNAs and nuclear shuttling [22, 23], and p27 is likely a cell-to-cell movement protein [22].

In this study, tobacco plants with suspected mild tobacco bushy-top disease were collected in three locations in Yunnan province. Full length genomes of the three new TBTV isolates from Jiangchuan (termed JC) and Midu (termed MD) were cloned and sequenced, revealing a distinctive difference from previously reported TBTV isolates: The first nucleotide of TBTV-JC, TBTV-MD-I and TBTV-MD-II is a guanylate compared with an adenylate reported for the other TBTV sequences. In addition, we compared these three new TBTV isolates with the five available TBTV sequences to study molecular diversity and recombination events among the isolates.

## Results

### Detection of TBTV, TVDV and TBTD-associated RNA in different sources of tobacco with tobacco bushy top disease

Total RNA was extracted from leaves of tobacco with suspected tobacco bushy top disease collected from three locations (JiangChuan county, MiDu county and BaoShan city) in China’s Yunnan Province. RT-PCR and subsequent sequencing revealed that TBTV was present only in the samples from JiangChuan and MiDu. None of the samples contained the newly reported TBTDaRNA (Additional file 1: Table S1).

### 5’-RACE and 3’-RACE of TBTV from JiangChuan and MiDu

**Fig. 1:**
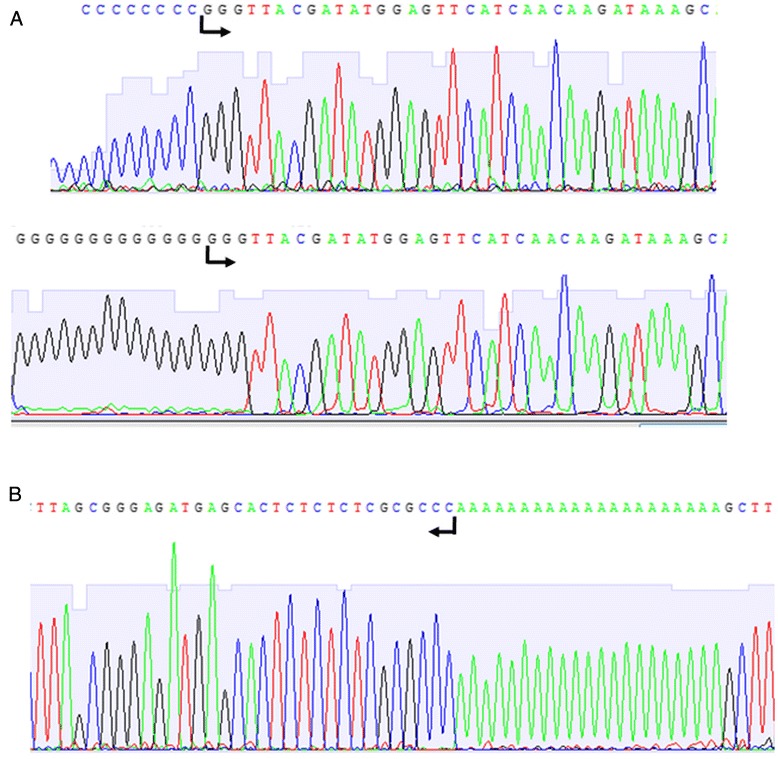
DNA sequencing for 5’-RACE and 3’RACE PCR products of TBTV-JC. A. 5’ RACE of TBTV-JC, Up: 5’RACE by adding poly (G) at the 5’ end of cDNA; Down: 5’RACE by adding poly (C) at the 5’ end of cDNA. B. 3’RACE of TBTV-JC. Fold arrows indicating the first (**a**) and last (**b**) nucleotide of TBTV-JC genome, respectively

To determine the full-length sequences of TBTV from JiangChuan (termed JC) and MiDu (termed MD), 5’-RACE and 3’-RACE were first performed to determine 5’ terminal and 3’ terminal sequences. The size of the 5’-RACE PCR product with either poly (C) or poly (G) at the 5’ end was approximately 500 bp (data not shown). Comparison of the sequencing results revealed that the first nucleotide of the JC isolate is a guanylate, with the sequence beginning with 5’-GGGUUACGAUAUGGAGUUCAUCAAC-3’ (Fig. 1a). The MD isolate also has the same sequences at the 5’ end of its genome. The first nucleotide of all previously reported TBTV isolates is an adenylate. Most Umbraviruses, as well as Necroviruses and Carmoviruses in the family *Tombusviridae,* also have 5’ terminal guanylates.

The size of the 3’-RACE PCR product of the JC and MD isolates was approximately 950 bp (data not shown). The 3’ terminal sequence of the JC and MD isolates is 5’-GGGAGAUGAGCACU***C***UCUCUCGCGCCC-OH-3’ (Fig. 1b). The underlined cytidylate differs from previous TBTV sequences, which contain an urydilate at this position. This substitution of U to C seems to deform the loop of the 3’ proximal stem-loop in TBTV (data not shown).

### Comparison of the sequences of the three new TBTV isolates and five previously published sequences

The full-length genomes of TBTV-JC (KM016224), TBTV-MD-I (KM016225) and TBTV-MD-II (KM067277) are 4152 nt in length, as previously reported for the other TBTV isolates. Comparison of the *in vitro* expression level of ORF1 (p35) and ORF1-frameshift protein (p98) for these isolates using wheat germ extracts (WGE) showed that that the translated levels of p35 and p98 for TBTV-JC, TBTV-MD-I and TBTV-MD-II (all mild isolates) was ~40 % of the TBTV-Ch level, which was cloned from a sample showing typical tobacco bushy top disease (Wang and Yuan, unpublished data). This suggests that attenuated expression of replicase components (p35 and p98) may be in part responsible for the mild pathogenicity of new TBTV isolates in the field.

Nucleotide sequence identities among TBTV-JC, TBTV-MD-I and TBTV-MD-II were 94.8 % to 97.3 % (Table 1). The highest nucleotide sequence identity among the 8 TBTV isolates was 98.9 % between TBTV-Ch and TBTV-YWSh, while the lowest was 89.0 % between TBTV-MD-II and TBTV-YWDu. The nucleotide sequence of TBTV-YWDu was the most different from the other 7 isolates, with identities ranging from 89.0 % to 90.9 %. Correspondingly, the nucleotide sequence identities among the other 7 isolates were 94.6 % to 98.9 % (Table 1).

**Table 1:** Nucleotide sequence identities (%) for TBTV-JC, TBTV-MD-I, TBTV-MD-II and previously reported TBTV isolates based on the full-length genome and 3’UTR sequence

	TBTV-JC	TBTV-MD-I	TBTV-MD-II	TBTV-Ch	TBTV-YWSh	TBTV-YYXi	TBTV-YLLi	TBTV-YWDu
	(KM016224)	(KM016225)	(KM067277)	(NC004366)	(FN256356)	(FN597051)	(FM242699)	(FM242700)
TBTV-JC		97.3	94.8	96.6	97.3	95.4	97.2	89.2
TBTV-MD-I	97.3		94.9	96.8	97.8	95.8	99.0	89.
TBTV-MD-II	94.7	94.9		95.5	96.2	94.6	95.4	89.0
TBTV-Ch	96.7	96.9	94.7		98.9	96.5	97.3	90.9
TBTV-YWSh	97.7	98.5	96.0	98.5		97.1	98.2	90.6
TBTV-YYXi	96.7	97.5	95.0	98.8	98.6		96.4	89.7
TBTV-YLLi	97.5	98.9	95.4	97.7	99.2	98.3		90.0
TBTV-YWDu	96.6	96.7	94.6	99.8	98.3	98.6	97.5	

Note:

1. The values were calculated using DNAMAN. Values above and below the diagonal shaded frames indicate the percentage of nucleotide sequence identity for the full-length genome and 3’UTR sequence, respectively

2. 5’UTR of TBTV isolates was 10 nt and only difference was the first nucleotide of guanylate or adenylate. The identities based on 5’UTR sequence among TBTV isolates is 90 % or 100 %

TBTV contains a 5’ UTR of 10 nt and 3’ UTR of 645 nt. The 5’UTR of the three new isolates only diverges in the 5’ ultimate nucleotide from the previously reported isolates. In the 3’UTR, identical residues ranged from 94.6 % to 99.8 % (Table 1), which was higher than values found for the full-length genome. In particular, TBTV-YWDu, whose full-length genome differed the most from the other isolates (sharing 89.0 % to 90.9 % identity), had 3’UTR sequences sharing 94.6 % to 99.8 % identity with the other isolates (Table 1).

The nucleotide sequence and amino acid identities of ORFs (p35, p98 [C-terminal], p26 and p27) were compared (Fig. 3a; Table 2). For all 8 TBTV isolates, the nucleotide sequence and amino acid identities are: p35, 75.0 % to 99.2 % and 73.7 % to 98.7 %; p98-C, 89.7 % to 99.5 % and 95.8 % to 99.8 %; p26, 95.8 % to 99.9 % and 89.9 % to 100 %; and p27, 95.5 % to 99.9 % and 95.5 % to 100 % (Table 2). Among the isolates, ORFs of TBTV-YWDu were the most divergent, particularly in p35 and p98-C regions. TBTV-MD-II and TBTV-YYXi also were more divergent than the other isolates (Table 2).

**Table 2:** Nucleotide sequence and amino acid identities (%) for TBTV-JC, TBTV-MD-I, TBTV-MD-II and previously reported TBTV isolates based on ORF1 (p35), p98-C, ORF3 (p26) and ORF4 (p27)

	TBTV-JC	TBTV-MD-I	TBTV-MD-II	TBTV-Ch	TBTV-YWSh	TBTV-YYXi	TBTV-YLLi	TBTV-YWDu
TBTV-JC		96.0	90.9	94.9	95.9	92.4	96.4	75.0
		(96.5)	(94.3)	(94.6)	(95.9)	(93.0)	(97.1)	(73.7)
TBTV-MD-I	97.3		90.5	94.3	95.9	92.5	99.2	75.7
	(98.9)		(92.7)	(92.7)	(94.0)	(92.4)	(98.7)	(73.7)
TBTV-MD-II	95.6	95.6		92.3	92.8	90.6	90.5	76.1
	(98.2)	(97.6)		(94.9)	(95.9)	(91.4)	(93.3)	(74.9)
TBTV-Ch	97.3	97.7	96.6		98.4	93.3	94.8	76.0
	(99.5)	(98.7)	(98.7)		(98.1)	(92.4)	(93.3)	(74.3)
TBTV-YWSh	97.5	98.0	96.9	99.5		94.4	96.4	76.7
	(99.3)	(98.9)	(98.7)	(99.8)		(94.0)	(94.6)	(74.3)
TBTV-YYXi	96.4	96.6	95.9	97.9	98.2		93.0	76.4
	(99.1)	(98.4)	(98.4)	(99.6)	(99.5)		(93.0)	(74.3)
TBTV-YLLi	96.9	98.4	96.8	98.0	98.3	97.4		75.9
	(98.9)	(99.3)	(98.0)	(99.1)	(99.3)	(98.7)		(74.0)
TBTV-YWDu	90.5	90.4	89.7	91.1	91.2	90.7	90.8	
	(96.4)	(95.8)	(95.8)	(96.5)	(96.5)	(96.4)	(95.8)	
TBTV-JC		98.6	97.3	97.2	98.3	95.8	98.5	96.9
		(96.2)	(93.7)	(92.4)	(95.8)	(89.9)	(96.2)	(92.0)
TBTV-MD-I	98.6		98.5	98.5	99.4	97.1	99.9	98.2
	(99.6)		(96.6)	(95.8)	(98.7)	(93.3)	(100)	(95.4)
TBTV-MD-II	97.4	98.5		97.8	98.7	96.9	98.6	97.5
	(98.8)	(99.2)		(94.1)	97.1)	(93.3)	(96.6)	(93.7)
TBTV-Ch	97.3	98.5	97.8		98.9	96.4	98.6	99.7
	(98.8)	(99.2)	(99.1)		(96.6)	(91.1)	(95.8)	(99.6)
TBTV-YWSh	98.4	99.5	98.8	98.9		96.9	99.6	98.6
	(99.2)	(99.6)	(99.6)	(99.6)		(93.3)	(98.7)	(96.2)
TBTV-YYXi	95.5	96.6	96.5	95.9	96.5		96.9	96.1
	(96.7)	(97.1)	(96.3)	(96.3)	(96.7)		(93.3)	(90.7)
TBTV-YLLi	98.5	99.9	98.6	98.6	99.6	96.5		98.3
	(99.2)	(99.6)	(99.6)	(99.6)	(100)	(96.7)		(95.4)
TBTV-YWDu	97.0	98.2	97.6	99.7	98.6	95.7	98.7	
	(98.0)	(98.4)	(98.4)	(99.2)	(98.8)	(95.5)	(98.8)	

Note:

1. The values were calculated using DNAMAN. Values in brackets indicate the percentage of amino acid, and values outside brackets indicate the percentage of nucleotide sequence

2. Values above and below the diagonal shaded frames in the up-half part indicate the percentage of ORF1 and p98-C respectively. Values above and below the diagonal shaded frames in the down-half part indicate the percentage of amino acid identity of ORF3 and ORF4 respectively

3. Position of ORF1 (p35), ORF3 (p26) and ORF4 (p27) is respectively 11–958 (315 aa), 2757–3470 (237 aa) and 2773–3507 (244 aa). p98 is expressed by frameshift mechanism from p35. Here C-terminal of p98 (p98-C) was analyzed for identities, since N-terminal of p98 has the same characteristic as p35

### Phylogenetic relationship among all TBTV isolates

**Fig. 2:**
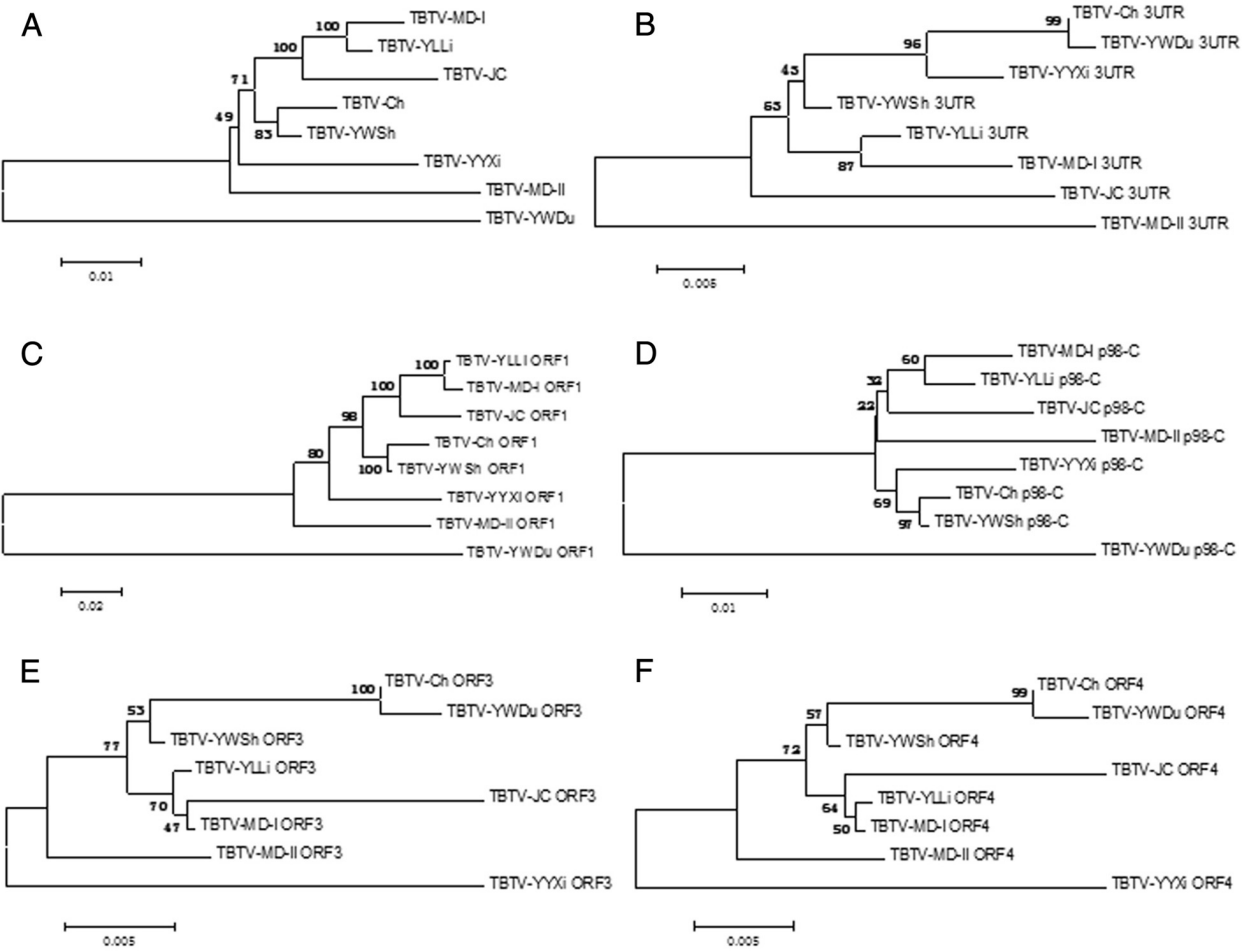
Phylogenetic trees of TBTV isolates. **a** Phylogenetic tree based on the full-length genome sequence of TBTV. **b** Phylogenetic tree based on the 3’UTR sequence of TBTV. **c** Phylogenetic tree based on the ORF1-coding nucleotide sequence of TBTV. **d** Phylogenetic tree based on the P98-C-coding nucleotide sequence of TBTV. **e** Phylogenetic tree based on the ORF3-coding nucleotide sequence of TBTV. **f** Phylogenetic tree based on the ORF4-coding nucleotide sequence of TBTV. All phylogenetic trees were constructed by the neighboring-joining (NJ) method and Komura 2-parameter method with bootstrap resampling (1000 replicates). The number at each branch of phylogenetic tree represents the bootstrap value (1000 replicates)

Phylogenetic trees were constructed based on the full-length genome, the 3’ UTR or ORFs-coding regions of TBTV. The distances of groups in phylogenetic trees of the full-length genome, ORF1 or p98-C were bigger than values of other regions (Fig. 2). It is suggested that RdRp-coding region was the most variable and mainly determined the divergent of TBTV isolates.

In the tree of the full-length genome, the 8 isolates were divided into three groups, with the first group only containing TVDV-YWDu (Fig. 2a). The second group contained TBTV-MD-II while the third group contained three sub-groups, one of which included one isolate of TBTV-YYXi; the other two sub-groups included 2 and 3 isolates respectively (Fig. 2a).

The phylogenetic trees based on ORFs or 3’ UTR have distinctive patterns. The tree based on ORF1 has the same grouping as the full-length genome (Fig. 2a and c). The other four trees have different pattern from the full-length genome. It is further confirmed that the divergence of ORF1-coding region is primarily correlated with the divergence of the full-length genome in TBTV.

In addition to the pattern of trees, there are some same clusters in different phylogenetic tree, i.e. the cluster containing TBTV-MD-I, TBTV-YLLi and TBTV-JC in trees of the full-length genome, ORF1, p98-C, ORF3 or ORF4-coding regions (Fig. 2a, c, d, e and f); the cluster containing TBTV-Ch and TBTV-YWSh in trees of the full-length genome, ORF1 or p98-C-coding regions (Fig. 2a, c and d ); and the cluster containing TBTV-Ch and TBTV-YWDu in trees of the 3’UTR, ORF3 or ORF4-coding regions (Fig. 2b, e and f). It is suggested that TBTV-MD-I, TBTV-YLLi and TBTV-JC have the nearest similarity except the 3’UTR, while TBTV-Ch and TBTV-YWSh have the nearest similarity in RdRp-coding region and TBTV-Ch and TBTV-YWDu have the nearest relationship in the 3’ half of genome including ORF3 and ORF4-coding regions and 3’UTR. All data of phylogenetic trees and molecular diversity assay suggested that different ORFs-coding regions and 3’ UTR were evolved separately.

### Recombination analysis of the TBTV isolates

**Fig. 3:**
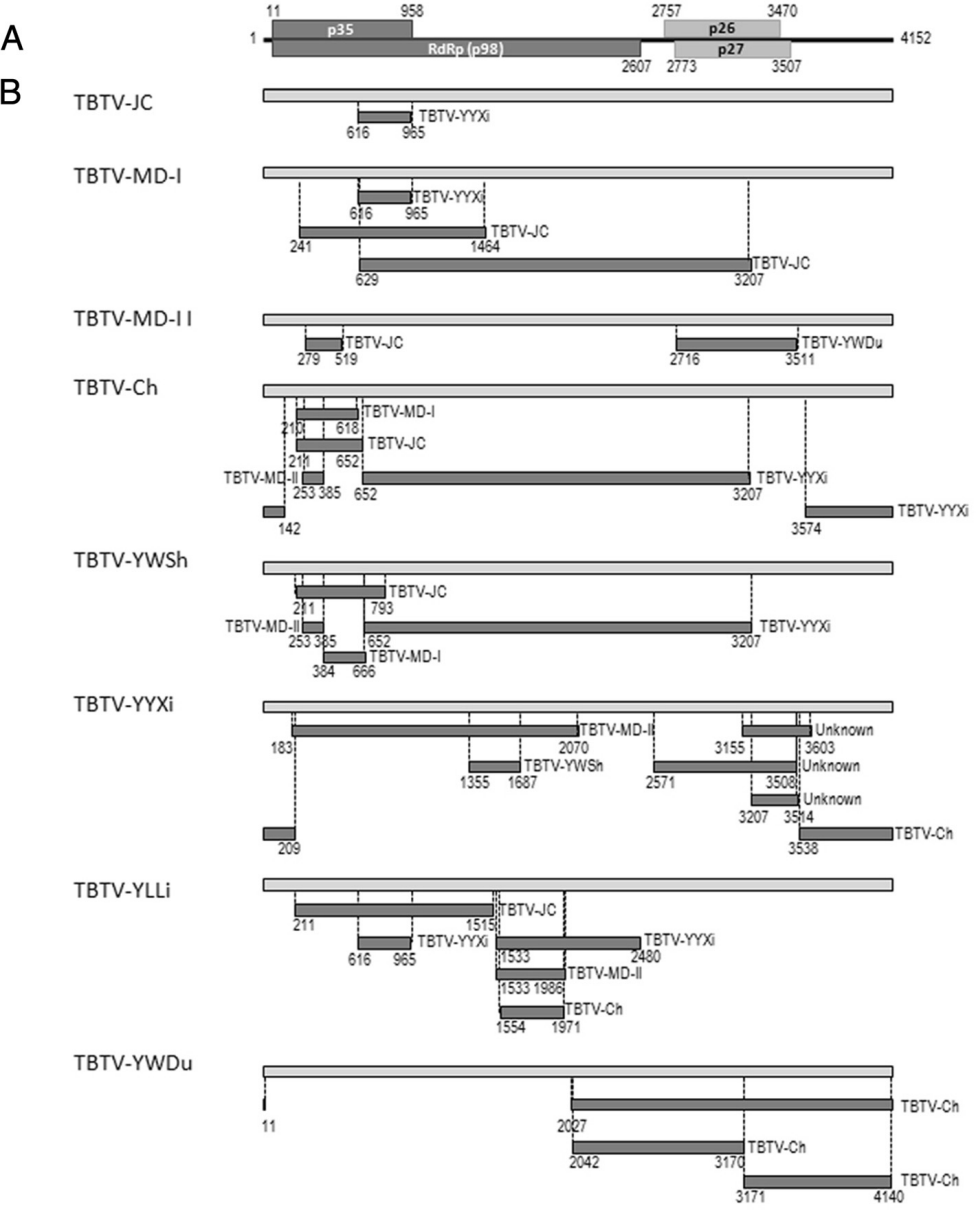
Analysis of possible recombination in different isolates of TBTV. **a** Genomic organization of TBTV. b. Summary of potential recombination in different isolates of TBTV. One events in TBTV-JC, three events in TBTV-MD-I, two events in TBTV-MD-II,five events in TBTV-Ch, four events in TBTV-YWSh, six events in TBTV-YYXi, five events in TBTV-YLLi and three events in TBTV-YWDu were detected. Dark bars in Fig. 3**b** indicating recombination regions with breakpoint positions and minor parent shown. Dot lines indicating breakpoints. Detailed information of recombination assay is provided in Table 3

To find potential recombination signals in the TBTV isolates, recombination analysis was performed using RDP4 program. Using six algorithms, 29 recombination events were detected in all 8 isolates (Fig. 3b and Table 3).TBTV-YYXi had 6 potential recombination signals, while TBTV-JC only had 1 potential recombination signal (Table 3).

**Table 3:** Summary of possible recombination events in TBTV isolates identified by RDP4

Event number	Recombinant Sequence(s)	Breakpoint position	Parental sequence (s)	Recombinant score	P-Value for the six detection methods in RDP4					
		Begin/End	Minor/Major		RDP	GENECONV	BootScan	MaxChi	Chimaera	SiScan
1	TBTV-JC	616/965	YYXi/MD-II	0.472	NS	NS	NS	8.76E-03	1.68E-02	4.07E-03
2	TBTV-MD-I	241/1464	JC/YYXi	0.469	2.71E-02	1.06E-02	1.11E-02	1.93E-05	4.78E-04	1.19E-09
3	TBTV-MD-I	616/965	YYXi/MD-II	0.472	NS	NS	NS	8.76E-03	1.68E-02	4.07E-03
4	TBTV-MD-I	629/3207	JC/YYXi	0.608	3.07E-02	NS	2.97E-02	1.65E-03	6.97E-03	5.17E-08
5	TBTV-MD-II	279/519	JC/Unknown (YYXi)	0.482	1.81E-03	NS	1.95E-02	3.48E-05	7.87E-06	3.56E-05
6	TBTV-MD-II	2716/3511	YWDu/Unknown (YYXi)	0.426	7.26E-04	NS	1.11E-02	1.22E-06	3.49E-06	NS
7	TBTV-Ch	210/618	MD-I/YYXi	0.426	NS	3.18E-02	2.76E-02	7.31E-03	3.32E-02	4.49E-06
8	TBTV-Ch	211/652	JC/YYXi	0.482	1.81E-03	NS	1.95E-02	3.48E-05	7.87E-06	3.56E-05
9	TBTV-Ch	253/385	MD-II/YLLi	0.419	NS	NS	NS	3.24E-02	NS	NS
10	TBTV-Ch	652/3207	YYXi/JC	0.608	3.07E-02	NS	2.97E-02	1.65E-03	6.97E-03	5.17E-08
11	TBTV-Ch	3574/142	YYXi/MD-I	0.461	1.23E-02	NS	1.37E-02	1.37E-02	1.62E-04	NS
12	TBTV-YWSh	211/793	JC/YYXi	0.483	1.04E-02	NS	1.00E-02	4.09E-02	1.23E-02	1.09E-06
13	TBTV-YWSh	253/385	MD-II/YLLi	0.419	NS	NS	NS	3.24E-02	NS	NS
14	TBTV-YWSh	384/666	MD-I/YYXi	0.426	NS	3.18E-02	2.76E-02	7.31E-03	3.32E-02	4.49E-06
15	TBTV-YWSh	652/3207	YYXi/JC	0.608	3.07E-02	NS	2.97E-02	1.65E-03	6.97E-03	5.17E-08
16	TBTV-YYXi	183/2070	MD-II/JC	0.593	2.95E-03	3.16E-02	8.25E-05	NS	NS	6.63E-05
17	TBTV-YYXi	1355/1687	YWSh/Unknown (MD-II)	0.420	NS	1.41E-02	NS	4.78E-02	NS	6.90E-06
18	TBTV-YYXi	2571/3508	Unknown (MD-II)/Ch	0.443	NS	NS	NS	1.53E-04	NS	NS
19	TBTV-YYXi	3155/3603	Unknown (MD-II)/ YLLi	0.615	1.03E-02	4.02E-04	2.49E-04	9.70E-04	7.29E-04	4.44E-05
20	TBTV-YYXi	3207/3514	Unknown(JC)/YWDu	0.841	3.26E-04	2.37E-02	NS	6.50E-04	1.63E-02	NS
21	TBTV-YYXi	3538/209	Ch/Unknown(MD-I)	0.534	NS	NS	NS	7.38E-06	4.47E-04	NS
22	TBTV-YLLi	211/1515	JC/YYXi	0.608	3.07E-02	NS	2.97E-02	1.65E-03	6.97E-03	5.17E-08
23	TBTV-YLLi	616/965	YYXi/MD-II	0.472	NS	NS	NS	8.76E-03	1.68E-02	4.07E-03
24	TBTV-YLLi	1533/1986	MD-II/MD-I	0.741	1.21E-08	3.18E-04	9.88E-09	8.14E-04	5.48E-04	NS
25	TBTV-YLLi	1533/2480	YYXi/MD-I	0.731	1.31E-03	NS	2.93E-02	3.86E-08	3.61E-05	NS
26	TBTV-YLLi	1554/1971	Ch/MD-I	0.611	NS	NS	3.56E-04	1.26E-03	NS	NS
27	TBTV-YWDu	2027/11	Ch/Unknown(MD-II)	0.632	3.34E-42	6.13E-42	1.08E-39	5.51E-19	7.16E-19	2.38E-23
28	TBTV-YWDu	2042/3170	Ch/Unknown(MD-II)	0.601	5.12E-23	1.81E-23	7.17E-25	3.99E-09	3.28E-11	1.92E-09
29	TBTV-YWDu	3171/4140	Ch/Unknown(MD-II)	0.545	3.34E-42	6.13E-42	1.08E-39	5.42E-06	5.80E-06	1.13E-14

NS: not significant

In all 29 potential recombination events, three recombination events (events 27, 28 and 29) detected in TBTV-YWDu had remarkable high degree of certainty with P-value of at least three algorithms <1 × 10^−6^ (Table 3). There are the other nine recombination events with a high degree of certainty due to recombinant score > 0.6, located at 629–3207 of TBTV-MD-I (event 4), 652–3207 of TBTV-Ch (event 10) and TBTV-YWSh (event 15), 3155–3603 and 3207–3514 of TBTV-YYXi (events 19 and 20), 211–1515, 1533–1986, 1533–2480 and 1554–1971 of TBTV-YLLi (events 22, 24, 25 and 26) (Table 3). In addition, remaining 17 recombination events have a fairly likelihood since recombinant score is between 0.4-0.6 (Table 3).

For all 8 TBTV isolates, there are three types based on the location of recombination events. The first type contained TBTV-YWDu, which only had recombinations at 3’ half of the genome; the second type contained TBTV-YLLi and TBTV-JC, which had recombinations at 5’ half of thegenome; and the third type contained 5 isolates of TBTV-MD-I, TBTV-MD-II, TBTV-Ch, TBTV-YWSh and TBTV-YYXi, which had recombinations throughout the genome (Fig. 3b).

In addition, there are same type of recombinant events with same locations and parents in different TBTV isolates including recombination located at 616–965 with parents TBTV-YYXi/TBTV-MD-II in TBTV-JC (event 1), TBTV-MD-I(event 3) and TBTV-YLLi (event 23), four recombinant events located at 253–385 with parents TBTV-MD-II/TBTV-YLLi and 652–3207 with parents TBTV-YYXi/TBTV-JC in TBTV-Ch (events 9 and 10) and TBTV-YWSh (events 13 and 15) (Fig. 3b and Table 3).

## Discussion

During the past decade, tobacco bushy top disease, which is mainly caused by TBTV and TVDV, underwent a sudden appearance, extreme virulence and degeneration of the epidemic, which is similar with the case of some extremely virulent RNA viruses (i.e. *Dengue type-2 virus, SARS coronavirus*, *Influenza A virus*) [24, 25]. Apart from the effective control on its aphid vectors, has the lethal mutagenesis of TBTV or TVDV contributed to the suppression of tobacco bushy top disease? In this study, we cloned three new isolates of TBTV showing mild pathogenicity in the field, and analyzed molecular diversity of these three new TBTV isolates plus the other five TBTV isolates previously reported in China during the outbreak of tobacco bushy top disease as well as phylogenetic relationship and possible recombination.

The three new isolates of TBTV (TBTV-JC, TBTV-MD-I and TBTV-MD-II) had a remarkable difference at the first nucleotide of G from five previously reported TBTV with the first nucleotide of A, while sizes of the genome and ORFs were same in all TBTV isolates. For ORFs encoded by the TBTV isolates, ORF3 and ORF4 was relative stable while ORF1 was the most variable based on the identities of ORFs-coding sequences among 8 TBTV isolates. This strong genetic variability was also identified in the RdRp-coding region of several plant viruses [26–29].

In all of 8 TBTV isolates, TBTV-YWDu had the most remarkable divergence from the other 7 isolates based on the identities of the full-length genome, ORF1or p98-C-coding sequences. TBTV-MD-II also showed difference from the other 7 TBTV isolates based on the identities of the 3’ UTR (Table 2). The remarkable divergence of TBTV-YWDu (Fig. 2a, c and d) and TBTV-MD-II (Fig. 2b) from the other TBTV isolates were also indicated by the phylogenetic trees. Two phylogenetic trees based on ORF3 and ORF4-coding sequences showed similar branches, which suggested that ORF3 of TBTV isolates underwent similar evolution as ORF4. Based on all data of molecular diversity and phylogentic tree, it is suggested that different ORFs-coding regions and the 3’ UTR in TBTV underwent separate evolution, and the diversity of ORF1-coding region mainly determined the diversity of full-length genome.

**Fig. 4:**
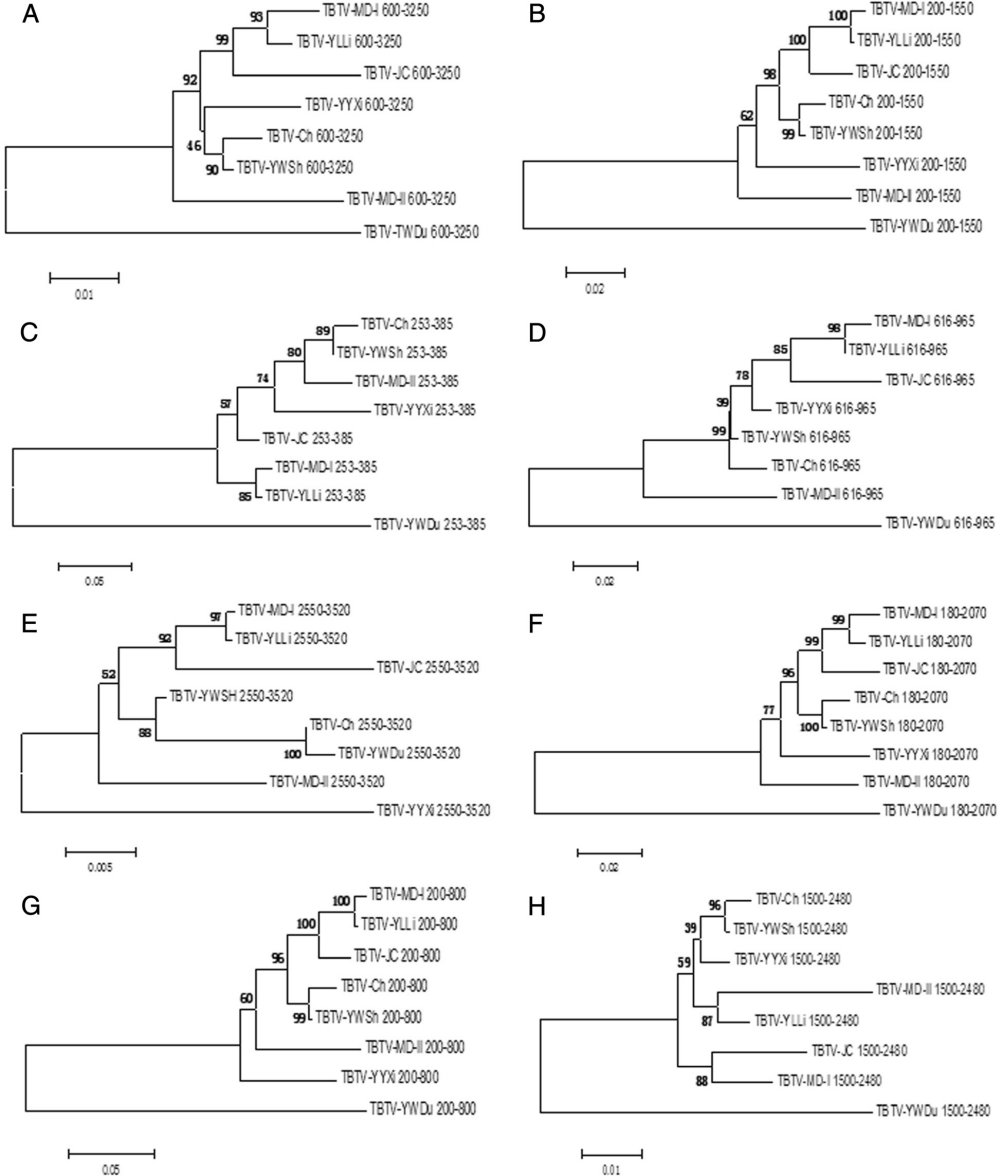
Phylogenetic trees of TBTV isolates. **a** Phylogenetic tree based on the fragment of 600–3250 in TBTV. **b** Phylogenetic tree based on the fragment of 200–1550 in TBTV. **c** Phylogenetic tree based on the fragment of 253–385 in TBTV. **d** Phylogenetic tree based on the fragment of 616–965 in TBTV. **e** Phylogenetic tree based on the fragment of 2550–3520 in TBTV. **f** Phylogenetic tree based on the fragment of 180–2070 in TBTV. **g** Phylogenetic tree based on the fragment of 200–800 in TBTV. **h** Phylogenetic tree based on the fragment of 1500–2480 in TBTV. All phylogenetic trees were constructed by the neighboring-joining (NJ) method and Komura 2-parameter method with bootstrap resampling (1000 replicates). The number at each branch of phylogenetic tree represents the bootstrap value (1000 replicates)

During evolution of the single strand RNA viruses, recombination is a major evolutionary way for an isolate to adapt to new environmental conditions and hosts [13–16]. In this study, recombination events were also analyzed among all 8 TBTV isolates. Total 29 potential recombination events were detected, in which three recombination events (events 27, 28 and 29) in TBTV-YWDu showed high reliability with P-value of at least three methods <1 × 10^−6^ (Fig. 3b and Table 3). These three recombination events in TBTV-YWDu were located within 3’ half and have same parents of TBTV-Ch/possible TBTV-MD-II, which is supported by the phylogenetic analysis. In phylogenetic trees based on ORF3, ORF4 and 3’UTR in 3’ half of TBTV genome, TBTV-YWDu and TBTV-Ch (the minor parent) formed into a cluster, while TBTV-MD-II (the possible major parent) belonged the other different groups (Fig. 2b, e, and f). However, TBTV-YWDu formed a separate group in phylogentic trees based on ORF1 and p98-C in 5’ half of genome (Fig. 2a and c), which implied why there is no recombination events in 5’ half of TBTV-YWDu. The other 9 recombination events (events 4, 10, 15, 19, 20, 22, 24, 25 and 26) seem to have a high degree of certainty due to recombination score > 0.6 (Table 3). Three recombination events (events 4, 10 and 15) with similar breakpoints (629–3207 in TBTV-MD-I, 652–3207 in TBTV-Ch and TBTV-YWSh) were supported by phylogenetic tree (Table 3 and Fig. 4a), in which TBTV-Ch and TBTV-YWSh formed into a branch with their minor parent TBTV-YYXi of events 10/15 and TBTV-MD-I formed into a branch with its minor parent TBTV-JC of event 4. Recombination event 22 was also supported by phylogenetic tree (Table 3 and Fig. 4b), in which TBTV-YLLi was formed into a branch with its minor parent TBTV-JC. Meanwhile, TBTV-JC was also the minor parent in recombination event 2 in TBTV-MD-I (recombination score is 0.469), which was also supported by phylogentic tree based on the fragment of 200–1550 (Fig. 4b). Three recombination events (events 24, 25 and 26) in TBTV-YLLi also showed some correlation with phylogenetic tree based on the fragment of 1500–2480 (Table 3 and Fig. 4h), in which TBTV-YLLi formed into a branch with its minor parent of event 24 and has near relationship with the minor parent TBTV-YYXi (event 25) or TBTV-JC (event 26). For two recombination events (events 19 and 20) in TBTV-YYXi, there is no correlation between recombination assay and phylogenetic tree based on ORF3 or ORF4-coding region (Fig. 2e and f), in which TBTV-YYXi formed into a separate group. It is suggested that events 19 and 20 are possible with uncertain minor parents (Table 3). For 17 recombination events with recombination score between 0.4-0.6, some of them also showed certain correlation with phylogenetic analysis. Three events (events 1, 3 and 23) with same breakpoints (616–965) in TBTV-JC, TBTV-MD-I and TBTV-YLLi were supported by phylogenetic tree based on the fragment of 616–965 (Fig. 4d), in which TBTV-JC, TBTV-MD-I and TBTV-YLLi formed into a branch having nearest relationship with their co-minor parent TBTV-YYXi. Two recombination events (events 9 and 13) with same breakpoints (253–385) in TBTV-Ch and TBTV-YWSh also showed some correlation with phylogenetic tree based on the fragment of 253–385 (Fig. 4c), in which TBTV-Ch and TBTV-YWSh formed into a branch having the nearest relationship with their co-minor parent TBTV-MD-II. For event 16 in TBTV-YYXi, clue of recombination was found from phylogenetic tree based on the fragment of 180–2070 (Fig. 4f), in which TBTV-YYXi formed into a branch separately derived from the branch of minor parent TBTV-MD-II. In addition, four recombination events (events 7, 8, 12 and 14) with similar breakpoints in TBTV-Ch and TBTV-YWSh also showed correlation with the phylogenetic based on the fragment of 200–800 (Fig. 4g), in which TBTV-Ch and TBTV-YWSh formed into a branch having the nearest relationship with other branch including their minor parents TBTV-JC and TBTV-MD-I. The other two recombination events (events 11 and 21) spanning 5’ and 3’ region in TBTV-Ch and TBTV-YYXi were also supported by the phylogenetic tree based on 3’UTR (Fig. 2b), in which TBTV-Ch and TBTV-YYXi along with TBTV-YWSh formed into a branch. In addition, clue of the event 17 in TBTV-YYXi can be found in the phylogenetic tree based on p98-C-coding sequences (Fig. 2d). Only three events (events 5, 6 and 18) were not supported by the corresponding phylogenetic trees (Fig. 4g and e). It is suggested that events 5, 6 and 18 are possible with uncertain minor parents (Table 3). All above data implied the inherent relationship between phylogenetic analysis and recombination assay. Based on the analysis on P-value, recombination score and the correlation with phylogenetic trees, 24 events within 29 possible recombination events are certainly true, while the other 5 events (events 5, 6, 18, 19 and 20) only have low-level likelihood.

For three new isolates of TBTV, they seemed to undergo distinctive evolution. Firstly, TBTV-JC and TBTV-MD-I had near relationship compared with TBTV-MD-II based on the sequence diversity, the phylogenetic analysis and the recombination assay. However, TBTV-MD-I have distinctive characteristic from TBTV-JC in recombination assay (Fig. 3b). TBTV-MD-II may have different ancestor since it always formed a different branch from the branch including TBTV-JC and TBTV-MD-I in all phylogenetic trees (Fig. 2). Although they seemed to undergo different evolution, they showed mild pathogenecity, which may due to following reasons. First, activity of RdRp was altered since RdRp-coding region is the most variable for TBTV. Second, the expression level of replicase components was lower than that of severe pathotype, which is partly confirmed by primary data of in vitro translation of p35 and p98 (Wang and Yuan, unpublished data). In addition, mutagenesis of TVDV may also partially cause the mild symptom of tobacco bushy top diseases, since TVDV could not support TBTV in some samples of tobacco bushy top disease such as sample from Baoshan city (Additional file 1: Table S1) and the other related data [7]. Further attention and effort are necessary to figure out the detailed mechanism on the lower expression of replicase components encoded by TBTV and the absence of interaction between TBTV and TVDV in nature.

## Conclusion

Three new TBTV isolates from tobacco bushy top samples with mild symptom were cloned. The first nucleotide of them is a guanylate instead of an adenylte reported in the other TBTV isolates. Identities and phylogenetic analyses indicated that variants ORFs and the 3’ UTR in TBTV were evolved separately. RdRp-coding region was the most variable among TBTV isolates and the divergence of ORF1 is mainly correlated with the divergence of the full-length genome. Frequent recombinations were detected among TBTV isolates. For three new TBTV isolates, they have different recombination pattern and may have different ancestors.

## Materials and methods

### Sources of virus samples

Tobacco plants showing suspected bushy top disease were collected from three locations (BaoShan city, JiangChuan county in Yuxi city, and MiDu county in Dali city) in Yunnan province of China in 2013. Baoshan city and Dali city are adjacent, and there are three another cities between them and Yuxi city. Disease samples were stored at −80 °C for further analysis.

### RT-PCR detection of TBTV, TVDV and TBTD-assocaited RNA

Total RNA was extracted from tobacco leaves using Trizol reagent (TransGen), and reverse-transcribed using M-MLV reverse transcriptase and oligonucleotides corresponding to TBTV, TVDV or TBTD-associated RNA (Additional file 1: Table S2). PCR amplification was performed using *Taq* DNA polymerase and pairs of oligonucleotides (Additional file 1: Table S2). For the detection of TBTV, two pairs of oligonucleotides, TB-2263-F/TB-3263-R and TB-667-F/TB-1630-R, were designed for RT-PCR assay. Accordingly, two pairs of oligonucleotides (TV-2728-F/TV-3458-R and TV-3454-F/TV-4166-R) for TVDV and one pair of oligonucleotides (TBTD-1507-F/TBTD-2016-R) for TBTD-associated RNA were designed for RT-PCR detection respectively (Additional file 1: Table S2). The PCR products were cloned into pMD18-T vector (TaKaRa) and sequenced by using M13 primers.

### 5’-RACE, 3’-RACE and full-length RT-PCR of TBTV

For 5’-RACE, the total RNA was reverse-transcribed by M-MLV reverse transcriptase using oligonucleotide TB-943-R and treated with a mixture of RNaseH and RNase A. After purification using a cDNA purification kit (TransGen), the cDNAs were extended using dCTP or dGTP and terminal deoxynucleotidyl transferase (TaKaRa) and then subjected to PCR amplification using Oligo(dG)-anchor primer/TB-943-R or Oligo(dC)-anchor primer/TB-943-R. The first-round PCR products were amplified using Anchor primer/TB-510-R. The final PCR products were cloned into vector pMD18-T and sequenced using M13 primers. At least three RT-PCR clones were sequenced to make sure the reliabilityof 5’-RACE result.

For 3’-RACE, total RNA and Oligo (dA)-linker were ligated with T4 RNA ligase (NEB). The oligo(dA)-linked RNAs were reverse-transcribed using Oligo (dT)-anti linker and then followed by PCR amplification using Oligo (dT)-anti linker/TB-3206-F. The final products were cloned into pMD18-T and sequenced using M13 primers. At least three RT-PCR clones were sequenced to make sure the reliabilityof 3’-RACE result.

For full-length RT-PCR of TBTV, total RNAs were reverse-transcribed by PrimeScript reverse transcriptase (Takara) using TBTV-3’-R. The cDNA were then subjected to PCR amplification using *LA Taq* (Takara) and oligonucleotides TBTV-5’-F and TBTV-3’-R. PCR products corresponding to full-length TBTV genomic RNA were cloned into pMD18-T and sequenced using M13 primers and TBTV-specific primers (detailed information not shown).

All primers used for 5’-RACE, 3’-RACE and full-length RT-PCR of TBTV were shown detailedly in Additional file 1: Table S2.

### Sequence assembly and alignment, construction of phylogenetic trees and Recombination analysis

Sequence assembly was accomplished by DNAMAN, which is also used to analyze the identities of nucleotide sequences or amino acids among TBTV isolates.

Phylogenetic tree was constructed using MEGA 5.0 software package [30] based on the neighbor-joining method and Kimura 2-parameter method. 1000 replicates of Bootstrap resampling was used to ensure the reliability of individual nodes in phylogenetic tree.

Recombination analysis was achieved using RDP4 [31]. Six methods including RDP, GENECONV, Bootscan, Maxchi, Chimaera and SiScan implemented in RDP4 was used to detect recombination events, likely parental isolates and recombination break points under default settings. If recombination event was supported by at least three methods with P-value <10^−6^ or the recombination score is above 0.6, this recombination event is certainly true. If the recombination score is between 0.4-0.6, this recombination event has a fair likelihood.

The complete sequences of three new TBTV isolates were deposited in Genbank with accession numbers of KM016224 (TBTV-JC), KM016225 (TBTV-MD-I) and KM067277 (TBTV-MD-II), and detailed sequence information are available from http://www.ncbi.nlm.nih.gov/nucleotide/. Sequences of the other 5 reported TBTV isolates (Genbank No: NC004366, FN256356, FN597051, FM242699 and FM242700) were also referenced from http://www.ncbi.nlm.nih.gov/nucleotide/.

## Additional file

Additional file 1: Table S1. Determination of TBTV, TVDV and TBTDaRNA in different sources of tobacco bushy top diseases. **Table S2**. Primers used in this paper.
