# Supplementary material for: Phylogenetic and recombination analysis of Tobacco bushy top virus in China
Source: Virol J. 2015 Jul 25;12:111. doi: 10.1186/s12985-015-0340-2 (PMC4514990; doi:10.1186/s12985-015-0340-2)
Supplement: Additional file 1: Table S1. — Determination of TBTV, TVDV and TBTDaRNA in different sources of tobacco bushy top diseases. Table S2. Primers used in this paper. [file 12985_2015_340_MOESM1_ESM.docx]

Table S1 Determination of TBTV, TVDV and TBTDaRNA in different sources of tobacco bushy top diseases

|  | Tobacco bushy top disease | | |
| --- | --- | --- | --- |
|  | JiangChuan | MiDu | BaoShan |
| TBTV | Yes | Yes | No |
| TVDV | Yes | Yes | Yes |
| TBTDaRNA | No | No | No |

Table S2 Primers used in this paper

| **Primer name** | **Sequences (5’⭢3’)** | **Source of Sequence**  **(Genbank No)** | **polarity** | **Purpose** |
| --- | --- | --- | --- | --- |
| TB-667-F | 5-CCCTCGCCCACTTGC-3 | TBTV (NC004366) | + | RT-PCR of TBTV |
| TB-1630-R | 5-GGGAGGCATCTAAACCCAC-3 | TBTV (NC004366) | - |  |
| TB-2263-F | 5-GCGCATGGAAAAATGAG-3 | TBTV (NC004366) | + |  |
| TB-3263-R | 5- GGGCTCGCGCCTGC-3 | TBTV (NC004366) | - |  |
| TV-2728-F | 5-TGTCTACGGATGAGCAAGTG-3 | TVDV (EF529624) | + | RT-PCR of TVDV |
| TV-3458-R | 5-CTGCAACTGAGTATGCTTGG-3 | TVDV (EF529624) | - |  |
| TV-3454-F | 5-TGCAGCGTTGGAAGTTCAAG-3 | TVDV (EF529624) | + |  |
| TV-4166-R | 5-GTTCCGTTGCCTTTATAGAGCA-3 | TVDV (EF529624) | - |  |
| TBTD-1507-F | 5-GGGATTCATGGAGACAGCTAG-3 | TBTDaRNA (EF529625) | + | RT-PCR of TBTDaRNA |
| TBTD-2016-R | 5-CTTCATCAACCCACGTTCGA-3 | TBTDaRNA (EF529625) | - |  |
| TB-943-R | 5-GGGCCCACATTGAAGGGC-3 | TBTV (NC004366) | - | 5’-RACE |
| TB-501-R |  | TBTV (NC004366) | - | 5’-RACE |
| Oligo(dG)-anchor primer | 5- GGCCACGCGTCGACTAGTAC(G)_16_-3 | none |  | 5’-RACE |
| Oligo(dC)-anchor primer | 5- GGCCACGCGTCGACTAGTAC(C)_16_-3 | none |  | 5’-RACE |
| Anchor primer | 5-GGCCACGCGTCGACTAGTAC-3 | none |  | 5’-RACE |
| Oligo (dA)-linker | 5-(A)_17_GCTTGAGCTCGAGTCCTCGTCACTCTGCTCACTGG-3 | none |  | 3’-RACE |
| Oligo (dT)-anti linker | 5-CCAGTGAGCAGAGTGACGAGGACTCGAGCTCAAGC(T)_17_-3 | none |  | 3’-RACE |
| TB-3206-F | 5-GAGCTACTTCATTGTATCG-3 | TBTV (NC004366) | + | 3’-RACE |
| TBTV-5’-F | 5-GGGTTACGATATGGAGTTCATCAAC-3 | Based on 5’ RACE | + | Cloning of full-length TBTV-JC or TBTV-MD |
| TBTV-3’-R | 5-GGGCGCGAGAGAGAGTGCTC-3 | Based on 3’ RACE | - |  |
